# Kisspeptin Signalling in the Hypothalamic Arcuate Nucleus Regulates GnRH Pulse Generator Frequency in the Rat

**DOI:** 10.1371/journal.pone.0008334

**Published:** 2009-12-16

**Authors:** Xiao-Feng Li, James S. Kinsey-Jones, Yewsong Cheng, Alice M. I. Knox, Yuanshao Lin, Nikoletta A. Petrou, Antonia Roseweir, Stafford L. Lightman, Stuart R. Milligan, Robert P. Millar, Kevin T. O'Byrne

**Affiliations:** 1 Division of Reproduction and Endocrinology, King's College London, London, United Kingdom; 2 Medical Research Council Human Reproductive Sciences Unit, The Queens Medical Research Institute, Edinburgh, United Kingdom; 3 Henry Wellcome Laboratory for Integrative Neuroscience and Endocrinology, University of Bristol, Bristol, United Kingdom; 4 Division of Medical Biochemistry, University of Cape Town, Cape Town, South Africa; University of Córdoba, Spain

## Abstract

**Background:**

Kisspeptin and its G protein-coupled receptor (GPR) 54 are essential for activation of the hypothalamo-pituitary-gonadal axis. In the rat, the kisspeptin neurons critical for gonadotropin secretion are located in the hypothalamic arcuate (ARC) and anteroventral periventricular (AVPV) nuclei. As the ARC is known to be the site of the gonadotropin-releasing hormone (GnRH) pulse generator we explored whether kisspeptin-GPR54 signalling in the ARC regulates GnRH pulses.

**Methodology/Principal Findings:**

We examined the effects of kisspeptin-10 or a selective kisspeptin antagonist administration intra-ARC or intra-medial preoptic area (mPOA), (which includes the AVPV), on pulsatile luteinizing hormone (LH) secretion in the rat. Ovariectomized rats with subcutaneous 17β-estradiol capsules were chronically implanted with bilateral intra-ARC or intra-mPOA cannulae, or intra-cerebroventricular (icv) cannulae and intravenous catheters. Blood samples were collected every 5 min for 5–8 h for LH measurement. After 2 h of control blood sampling, kisspeptin-10 or kisspeptin antagonist was administered via pre-implanted cannulae. Intranuclear administration of kisspeptin-10 resulted in a dose-dependent increase in circulating levels of LH lasting approximately 1 h, before recovering to a normal pulsatile pattern of circulating LH. Both icv and intra-ARC administration of kisspeptin antagonist suppressed LH pulse frequency profoundly. However, intra-mPOA administration of kisspeptin antagonist did not affect pulsatile LH secretion.

**Conclusions/Significance:**

These data are the first to identify the arcuate nucleus as a key site for kisspeptin modulation of LH pulse frequency, supporting the notion that kisspeptin-GPR54 signalling in this region of the mediobasal hypothalamus is a critical neural component of the hypothalamic GnRH pulse generator.

## Introduction

Inactivating mutations of the kisspeptin receptor (GPR54) in humans are associated with a failure to progress through puberty and adult infertility (hypogonadotropic hypogonadism) [1], [2]. Kisspeptin administration stimulates GnRH or LH secretion in various species including mice [3], rats [4], [5], sheep [6], monkeys [7] and Humans [8]. The recent development of selective kisspeptin antagonists [9] has facilitated investigation of the role of endogenous kisspeptin in the control of the hypothalamo-pituitary-gonadal axis. Central administration of the kisspeptin antagonist, peptide 234, inhibited the post-castration rise in LH secretion in mice, blunted the LH response to exogenous kisspeptin in rats, suppressed LH pulse frequency and amplitude in ewes and suppressed GnRH pulses in monkeys [9]. However, the precise neural site of action remains to be established.

Kisspeptin perikarya are located in two discrete hypothalamic regions in rodents; the anteroventral periventricular nucleus (AVPV) and the arcuate nucleus (ARC) [3], [10]. The AVPV sends projections to the GnRH-rich medial preoptic area (mPOA) [11] where kisspeptin fibers appear in close apposition to GnRH perikarya [12]; the latter express GPR54 mRNA and show c-FOS expression after kisspeptin administration [13].

Kisspeptin neurons in the AVPV and ARC of rodents are the target for estrogen positive and negative feedback action respectively, since *Kiss1* mRNA expression is increased in the former and decreased in the latter nucleus in response to the steroid [14]. Further, the LH surge is blocked by injection of metastin antibodies into the mPOA [10] and GPR54- or kisspeptin-null mice fail to show LH surges [15]. In contrast to the AVPV, little is known about the role of kisspeptin-GPR54 signalling in the ARC in the regulation of gonadotropic hormone secretion in rodents. Nevertheless, the ARC is considered to be the prime location of the GnRH pulse generator in the rat [16], in common with other species including primates [17].

To further explore the relationship between kisspeptin-GPR54 signalling in the control of GnRH pulse generator activity, we examined the effects of icv administration of kisspeptin antagonist on pulsatile LH secretion in the rat. To test the hypothesis that kisspeptin-GPR54 signalling in the ARC plays a critical role in controlling GnRH pulse generator activity, we examined the effects of kisspeptin or kisspeptin antagonist microinfused into this brain region on pulsatile LH secretion and compared the response to microinfusion into the mPOA.

## Materials and Methods

### Animals and Surgical Procedures

Adult female Sprague Dawley rats (220–250 g) obtained from Charles River (Manston, UK) were housed under controlled conditions (12∶12-h light/dark cycle with lights on at 0700 h; temperature at 22±2°C) and provided with food and water *ad libitum*. All animal procedures were undertaken in accordance with the United Kingdom Animals (Scientific Procedures) Act, 1986, under Licence # 70/6237. Experimental procedures on animals were also approved by the King's College London Ethical Review Panel Committee. Animals were allowed to habituate to the animal unit for one week before initiation of experimentation. All surgical procedures were carried out under ketamine anaesthesia (100 mg/kg i.p.; Pharmacia and Upjohn Ltd., Crawley, UK) and Rompun (10 mg/kg i.p.; Bayer, Leverkusen, Germany).

Rats were bilaterally ovariectomized and implanted with a Silastic capsule (inner diameter, 1.57 mm; outer diameter, 3.18 mm; Sanitech, Havant, UK), filled to a length of 25 mm with 17β-estradiol (E_2_) (Sigma-Aldrich, Poole, UK) dissolved at a concentration of 20 µg/ml arachis oil (Sigma-Aldrich). The E_2_-containing capsules produced circulating concentrations of E_2_ within the range observed during the diestrous phase of the estrous cycle (∼38.8±1.2 pg/ml) as previously described [18].

**Figure 1:**
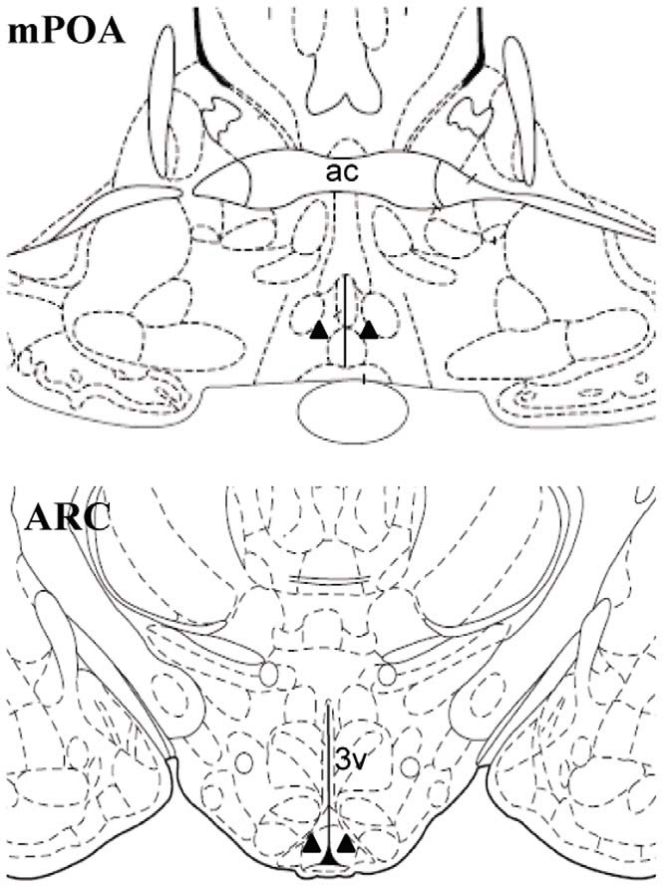
Schematic illustration of the intra-cerebral microinjection sites. Bilateral cannulae were positioned in the medial preoptic area (mPOA) at bregma −0.26 mm or the hypothalamic arcuate nucleus (ARC) at bregma −3.30 mm according to the rat brain atlas of Paxinos and Watson (19). Closed triangles represent the location of the cannulae tips. Ac, anterior commissure; 3v, third cerebral ventricle.

To evaluate the inhibitory effect of the selective kisspeptin antagonist on pulsatile LH secretion, a group of rats was implanted with an icv guide cannula (22 gauge; Plastics One, Roanoke, VA, USA) directed towards the left lateral ventricle, the co-ordinates for implantation being 0.6 mm lateral and 1.5 mm posterior to bregma, and 4.5 mm below the surface of the dura. The guide cannula was secured using dental cement (Dental Filling Ltd., Swindon, UK), and fitted with a dummy cannulae (Plastics One) to maintain patency. To identify the neural site for kisspeptin-GPR54 signalling to control pulsatile LH secretion, a separate group of rats was implanted with bilateral guide cannulae (22-gauge; Plastics One) directed at the GnRH-rich areas of the mPOA or the ARC for microinfusion of kisspeptin or its antagonist. The coordinates for implantation for the mPOA and ARC cannulae were: 0.5 mm lateral, 0.26 mm posterior to bregma and 8.6 mm below the surface of the dura, and 0.4 mm lateral, 3.3 mm posterior to bregma, and 10.2 mm below the surface of the dura, respectively [19]. Schematic illustration of the microinjection sites is shown in Figure 1. The guide cannulae were secured using dental cement (Dental Filling Ltd.) and fitted with dummy cannulae (Plastics One). All brain cannulae were implanted at the time of ovariectomy. After a 10-day recovery period, the rats were fitted with two indwelling cardiac catheters via the jugular veins. The catheters were exteriorised at the back of the head and secured to a cranial attachment; the rats were fitted with a 30-cm-long metal spring tether (Instec Laboratories Inc., Boulder, CO, USA). The distal end of the tether was attached to a fluid swivel (Instec Laboratories), which allowed the rat freedom to move around the enclosure. Experimentation commenced 3 day later. Correct cannula placement in the mPOA or ARC was confirmed by injection of 500 nl India ink through the internal guide cannulae followed by microscopic inspection of 30 µm frozen brain sections. Only data from animals with correct cannula placement were analysed.

### Icv Infusion of Kisspeptin or Antagonist and Pulsatile LH Secretion

On the morning of experimentation, a single internal injection cannula (Plastics One) with extension tubing, preloaded with a selective kisspeptin antagonist [9] (peptide 234, 7.5 nmol in 12 µl aCSF; N = 6) or aCSF (12 µl aCSF; N = 5) was inserted in the guide cannula, extending 1.0 mm beyond its tip to reach the left ventricle. The distal end of the tubing was extended outside of the animal cage and attached via a dual-channel swivel (Instec Laboratories) connected to a 25 µl Gastight Hamilton syringe (Waters International, UK) secured in a microinjection syringe pump (2000 Syringe Pump; Harvard Apparatus, Massachusetts, USA) programmed to deliver 4 µl/h for 3 h, thus allowing remote infusion without disturbing the rat during the experiment. Rats were also attached via one of the two cardiac catheters to a computer-controlled automated blood sampling system, which allows for the intermittent withdrawal of small blood samples (25-µl) without disturbing the animals. Once connected, animals were left undisturbed for 1 h before sampling commenced. Blood sampling commenced between 0900 and 1000 h when samples were collected every 5 min for 8 h for LH measurement. After removal of each 25-µl blood sample, an equal volume of heparinized saline (5 U/ml normal saline; CP Pharmaceuticals Ltd., Wrexham, UK) was automatically infused into the animal to maintain patency of the catheter and blood volume. After 2 h of controlled blood sampling, kisspeptin antagonist or aCSF were infused in the relevant animals over a period of 3 h. Blood sampling continued throughout the experiment. Blood samples were frozen at −20°C for later assay to determine LH concentrations.

### Intra-ARC or Intra-mPOA Infusion of Kisspeptin-10 and Pulsatile LH Secretion

For the administration of kisspeptin-10, bilateral injection cannulae (Plastics One) with extension tubing preloaded with different doses of kisspeptin-10 (Sigma-Aldrich) (1 pmol, N = 6; 10 pmol, N = 8; 100 pmol, N = 8) in 400 nl aCSF per brain loci (intra-ARC or mPOA) or aCSF (400 nl; N = 6 for the ARC and N = 7 for the mPOA) were inserted into the guide cannulae, extending 1.0 mm beyond its tip to reach the site of the brain nuclei. The distal end of the tubing was extended outside of the animal cage to allow remote infusion without disturbing the rat during the experiment. The automated blood sampling system was set up in the same way as described above for the collection of 25-µl blood samples every 5 min, but only for 5 h. All treatments were given by injection over 5 min after 2 h of the onset of blood sampling.

### Intra-mPOA or Intra-ARC Infusion of Kisspeptin Antagonist and Pulsatile LH Secretion

For the infusion of kisspeptin antagonist into the mPOA or ARC, internal cannulae loaded with the antagonist or aCSF were set up in the same way as described above. Rats were administrated with 10 pmol (mPOA, N = 8; ARC, N = 7 for the ARC) or 50 pmol (mPOA, N = 8; ARC, N = 8) kisspeptin antagonist in 500 nl aCSF over the period of 5 min after 2 h of controlled blood sampling and then the same dosage was repeated on two further occasions at an interval of 30 min. Control animals received 500 nl aCSF only with the same regimen (mPOA, N = 7; ARC, N = 6). Blood sampling procedures were set up as described above for LH measurement.

### RIA for LH Measurement

A double-antibody RIA supplied by the National Institute of Diabetes and Digestive and Kidney Diseases was used to determine LH concentration in the 25-µl whole blood sample [20]. The sensitivity of the assay was 0.093 ng/ml. The intraassay variation was 8.5% and the interassay variation was 9.5%.

### Data and Statistical Analysis

Detection of LH pulse frequency and amplitude was established by use of the algorithm ULTRA [21]. Two intra-assay coefficients of variation of the assay were used as the reference threshold for the pulse detection. The inhibitory effect of infusion of kisspeptin antagonist (icv) on pulsatile LH secretion was analysed by comparing the mean LH pulse interval before, during and after treatments. The period duration in min of the 2 h pre-, 3 h during and the 3 h post-treatment was divided by the number of LH pulses detected in each of these periods to give the appropriate LH pulse interval. When there were no LH pulses evident during the 3 h treatment period, the LH pulse interval assigned to this period was taken as the interval from the onset of treatment to the first LH pulse in the 3 h post-treatment period. In addition, for the icv kisspeptin antagonist treated animals, the mean LH interpulse interval in the pre-treatment period was compared with the post-treatment period from the time of resumption of LH pulses onwards. The significance of the effect of icv infusion of kisspeptin antagonist on LH pulse intervals was also compared with their control animals injected with aCSF alone at the same time points. The effect of icv administration of kisspeptin antagonist on LH pulse amplitude was compared before and during treatment only. The effect of kisspeptin-10 administration into the ARC or mPOA on LH secretion was calculated by comparing the area under the LH profile in the 2 h baseline control blood sampling period to the 1 h period after administration of drug using SigmaPlot version 11 (Systat Software Inc., San Jose, CA, USA). The inhibitory effect of administration of kisspeptin antagonist into the mPOA or ARC on LH pulses was calculated by comparing the mean LH pulse interval before, 1^st^ h and 3^rd^ plus 4^th^ h after treatment onset in each group. For intra-mPOA and intra-ARC administration of kisspeptin antagonist, LH pulse amplitude was compared before and during the 1^st^ h after treatment onset in each group. Values given in the text and figures represent mean ± SEM. Comparisons between groups were made by subjecting data to ANOVA and Dunnett's test.

## Results

### Effect of Icv Administration of Kisspeptin Antagonist on Pulsatile LH Secretion

**Figure 2:**
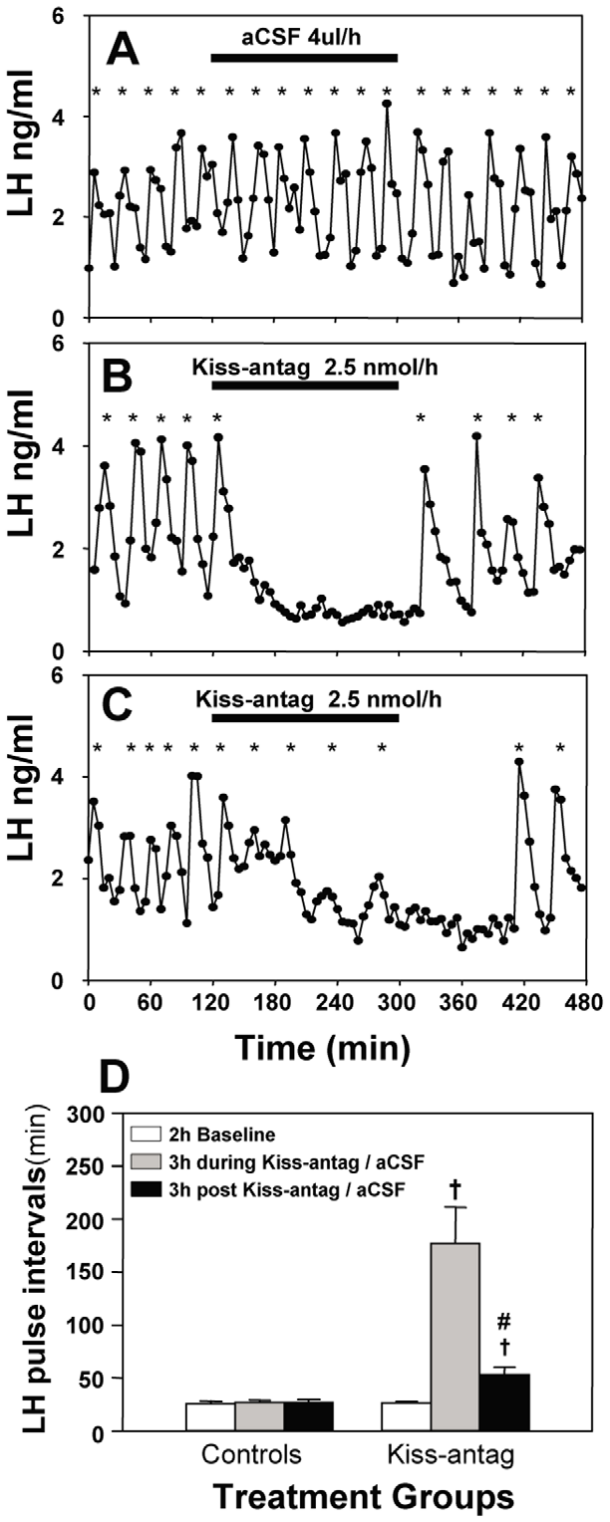
Effect of intracerebroventricular (icv) administration of kisspeptin antagonist (Kiss-antag) on LH pulse frequency. Representative examples illustrating the effects of continuous icv infusion of (A) aCSF (4 µl/h for 3 h) or (B and C) Kiss-antag (2.5 nmol/h for 3 h) in ovariectomized 17βestradiol-replaced rats. Pulsatile LH secretion was either completely suppressed during the period of Kiss-antag infusion (B) or LH pulse interval was significantly prolonged by Kiss-antag (C). D, Summary showing the inhibitory effect of Kiss-antag on pulsatile LH secretion. ^†^P<0.001 versus aCSF control group at the same time point. ^#^P<0.001 versus Kiss-antag treated group during the time of infusion; N = 5–6 per group. *LH pulse.

Pulsatile LH secretion representing normal activity of the GnRH pulse generator was detected in both kisspeptin antagonist and artificial cerebrospinal fluid (aCSF) treated animals during the 2-h baseline control period, with no significant difference in LH pulse frequency between experimental groups. Kisspeptin antagonist profoundly suppressed pulsatile LH secretion (Fig. 2*B*–*D*; P<0.05). LH pulses were effectively suppressed immediately after the onset of antagonist (2.5 nmol/h) infusion in 4 out of 6 rats and all 4 of these animals showed a complete absence of LH pulses during the period of kisspeptin antagonist infusion (Fig. 2*B*). Of the remaining animals, one displayed a single LH pulse at 50 min from the onset of kisspeptin antagonist infusion, and the second showed a gradual reduction in LH pulse frequency, though of smaller amplitude during the infusion (Fig. 2*C*; Table 1). The pulsatile pattern of LH secretion generally returned within 1–2 h after the end of the kisspeptin antagonist infusion, albeit of reduced frequency upon resumption of LH pulses (pre-treatment period versus post-treatment period from the time of resumption of LH pulses onwards: 26.42±1.42 versus 41.01±4.30 LH interpulse interval in minutes respectively; mean ± SEM; N = 5–6; P<0.05). Central administration of aCSF did not affect pulsatile LH secretion (Fig. 2*A and D*).

**Table 1:** Effect of intracerebroventricular (icv), intra-medial preoptic area (mPOA) or intra-arcuate nucleus (ARC) administration of kisspeptin antagonist, peptide 234 (Kiss-antag), on LH pulse amplitude (ng/mg) in ovariectomized 17β-estradiol-replaced rats.

Treatment groups	n	Baseline	During	Post
**ICV**				
aCSF (4 ul/h for 3 h)	5	1.90±0.22	2.01±0.30	2.19±0.33
Kiss-antag (2.5 nmol/h for 3 h)	6	1.91±0.41	1.07±0.25*	2.61±0.50
**Intra-POA**				
aCSF (0.5 ul ×3)	6	1.97±0.29	1.99±0.32	2.03±0.34
Kiss-antag (10 pmol ×3)	8	1.92±0.17	2.07±0.16	1.97±0.15
Kiss-antag (50 pmol ×3 )	7	1.96±0.29	1.98±0.36	1.95±0.25
**Intra-ARC**				
aCSF (0.5 ul ×3)	5	1.73±0.33	1.75±0.37	1.80±0.39
Kiss-antag (10 pmol ×3)	6	1.77±0.54	1.81±0.46	1.87±0.49
Kiss-antag (50 pmol ×3)	6	1.92±0.27	1.88±0.38	2.04±0.37

*2 rats continued to show LH pulses during kisspeptin antagonist infusion**/**not significant from baseline value (P = 0.28).

LH pulse amplitude (Mean ± SEM) is indicated before, during and post administration of antagonist or artificial cerebrospinal fluid (aCSF).

### Effects of Intra-ARC and Intra-mPOA Administration of Kisspeptin-10 on LH Secretion

**Figure 3:**
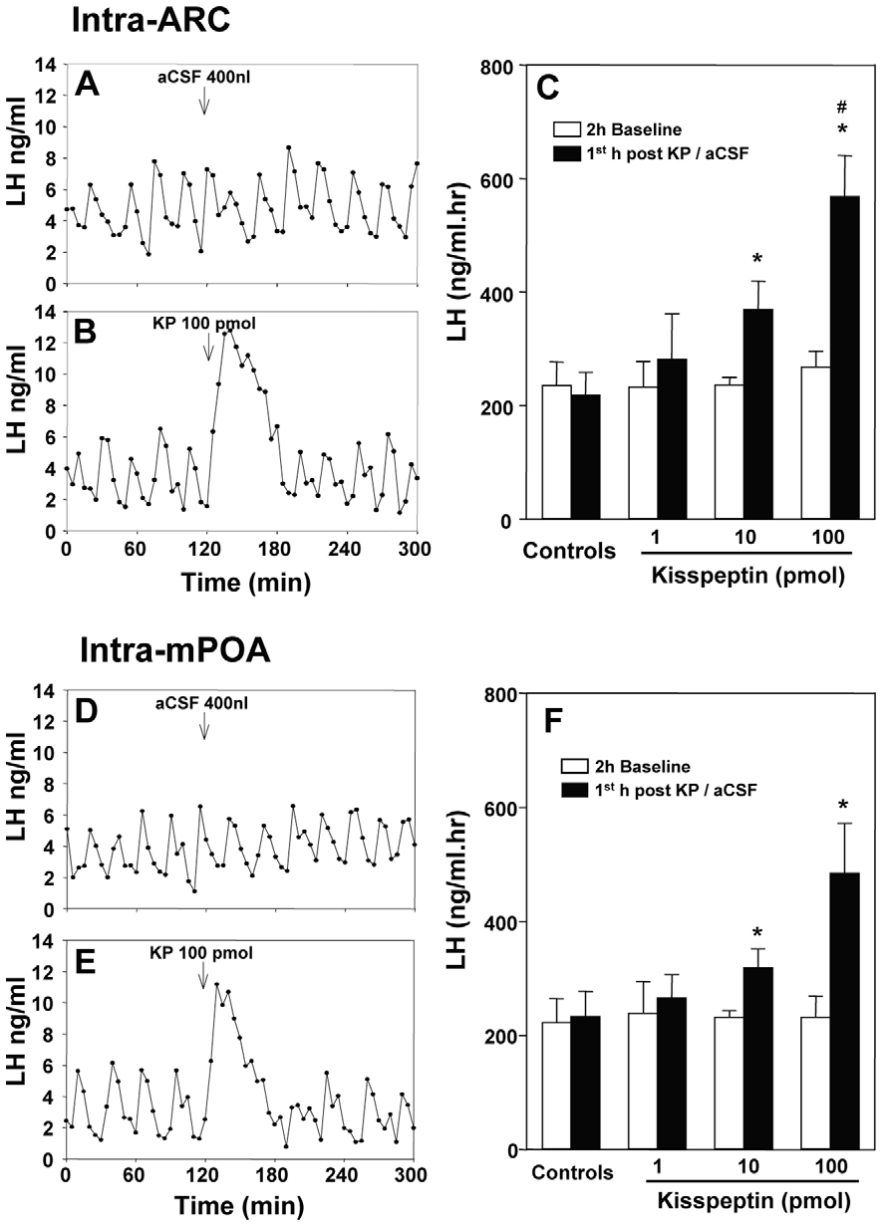
Effect of intra-arcuate nucleus (ARC) and intra-medial preoptic area (mPOA) administration of kisspeptin-10 (KP) on LH secretion. Representative examples illustrating the effects of intra-ARC infusion of (A) 400 nl aCSF or (B) 100 pmol KP in ovariectomized 17βestradiol-replaced rats. C, Summary showing the effect of KP on LH secretion, calculated by comparing the mean area of under LH profile 2 h before with 1 h after its administration. Representative examples illustrating the effects of intra-mPOA infusion of (D) 400 nl aCSF or (E) 100 pmol KP in ovariectomized 17βestradiol-replaced rats. F, Summary showing the effect of intra-mPOA KP on LH secretion. LH secretion was dramatically increased immediately after KP treatment in both nuclei, which lasted about 1 h in most experimental animals. *P<0.05 versus aCSF control group at the same time point. ^#^P<0.05 versus 10 pmol KP treatment group at the same time point; N = 5–7 per group.

Both intra-ARC and intra-mPOA administration of kisspeptin-10 resulted in a dose-dependent increase in circulating levels of LH that lasted approximately 1 h before recovering to a normal pulsatile pattern of LH secretion (Fig. 3*B and 3E*; P<0.05). The mean value of area under the LH profile during the first hour of kisspeptin-10 infusion was significantly greater in the 100 pmol than in the 10 pmol kisspeptin-10 treated group (Fig. 3*C and F*; P<0.05). Control intra-nuclear injection of aCSF had no effect on LH secretion (Fig. 3*A and 3D*).

### Differential Effect of Kisspeptin Antagonist Administration into the mPOA and the ARC on Pulsatile LH Release

**Figure 4:**
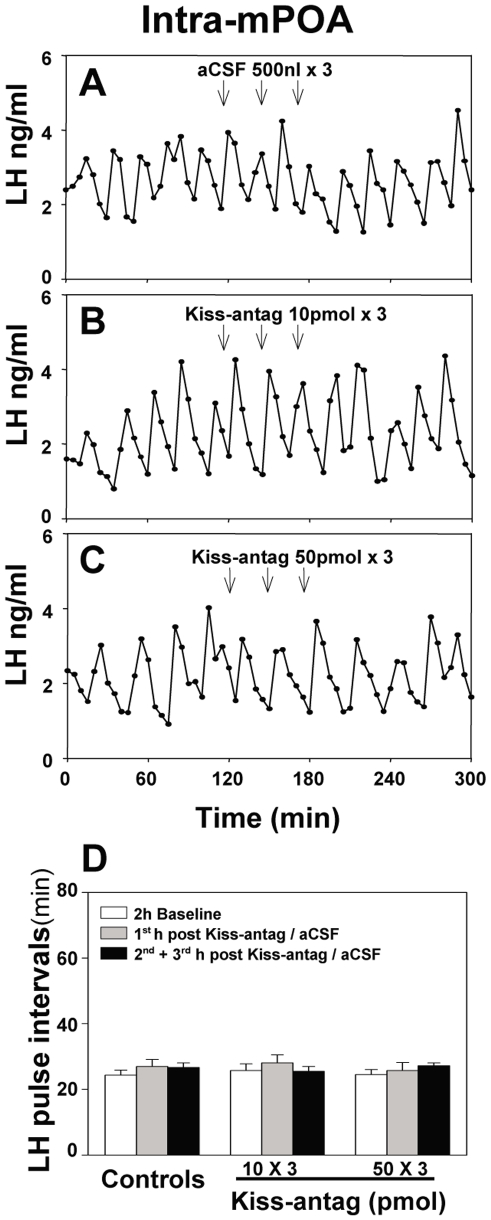
Effect of intra-medial preoptic area (mPOA) administration of kisspeptin antagonist (Kiss-antag) on pulsatile LH secretion. Representative examples illustrating the effects of intra-mPOA injection of (A) of 500 nl aCSF (3 injections at 30 min intervals), (B) 10 pmol Kiss-antag (3 injections at 30 min intervals) or (C) 50 pmol Kiss-antag (3 injections at 30 min intervals) in ovariectomized 17βestradiol-replaced rats. D, Summary showing there was no inhibitory effect of Kiss-antag on LH pulse interval. N = 6–8 per group.

**Figure 5:**
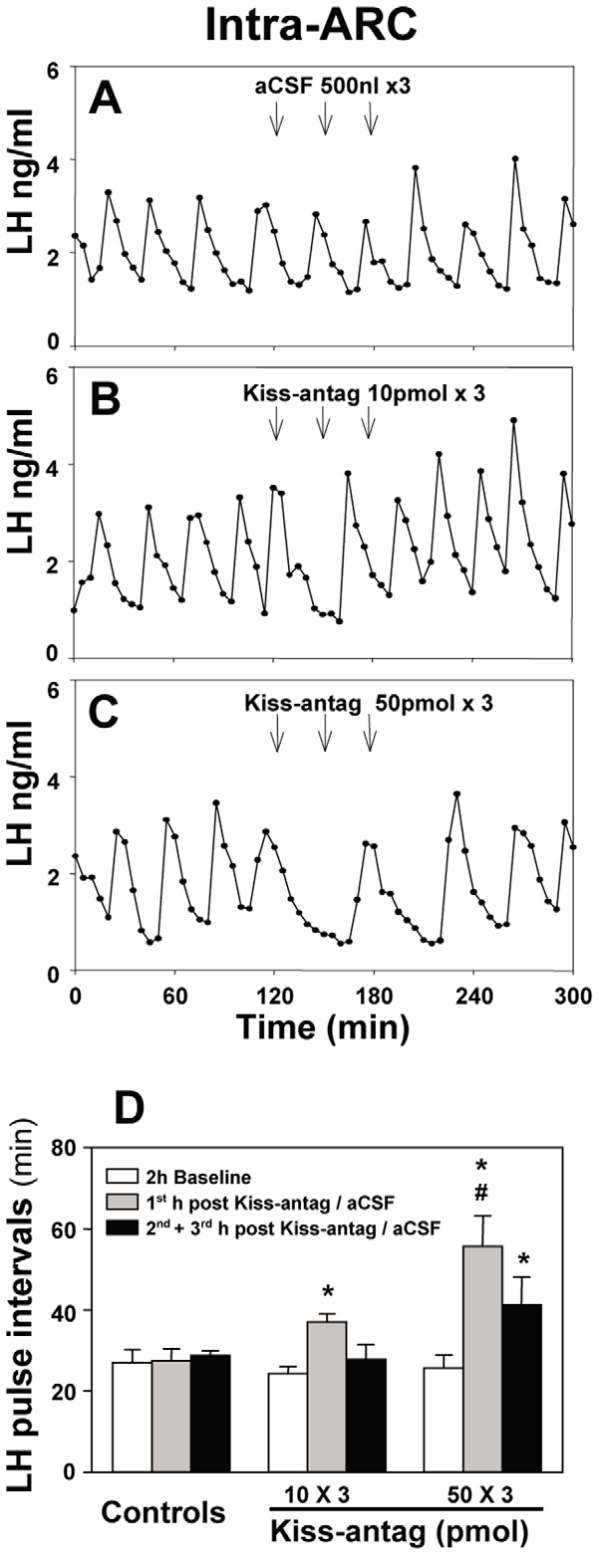
Effect of intra-arcuate nucleus (ARC) administration of kisspeptin antagonist (Kiss-antag) on pulsatile LH secretion. Representative examples illustrating the effects of intra-ARC injection of (A) of 500 nl aCSF (3 injections at 30 min intervals), (B) 10 pmol Kiss-antag (3 injections at 30 min intervals) or (C) 50 pmol Kiss-antag (3 injections at 30 min intervals) in ovariectomized 17βestradiol-replaced rats. D, Summary showing the inhibitory effect of Kiss-antag on LH pulse frequency. *P<0.05 versus aCSF control group at the same time point. ^#^P<0.001 versus Kiss-antag (10 pmol ×3) at the same time point; N = 5–6 per group.

To identify the site of action for the inhibitory effect of the kisspeptin antagonist on pulsatile LH secretion, 10 pmol or 50 pmol of the antagonist was administered into the mPOA or ARC as 3 injections at 30 min intervals starting after 2 h baseline control blood sampling. (Multiple injections were employed as continual infusion was impractical in this model). Administration of kisspeptin antagonist into the mPOA did not affect LH pulse frequency (Fig. 4*B*–*D*) or LH pulse amplitude (Table 1). However, intra-ARC administration of 10 pmol of antagonist suppressed LH pulses, resulting in an immediate prolongation of LH pulse interval (Fig. 5*B*–*D*; P<0.05) compared with the aCSF controls, which showed no change during the experimental period (Fig. 5*A and D*). The suppression of LH pulse frequency was further enhanced in response to the higher dose of antagonist (50 pmol ×3) (Fig. 5*C and D*; P<0.001). LH pulse amplitude was not affected by kisspeptin antagonist administration into the ARC (Table 1).

## Discussion

The results of this study demonstrate that administration of a selective kisspeptin antagonist profoundly suppressed pulsatile LH secretion in the rat. They support findings in the rhesus monkey where stalk-median eminence perfusion of the antagonist apparently suppressed GnRH pulses and in the ewe where icv infusion of the antagonist apparently suppressed LH pulse amplitude [9]. However, as in these previous studies, it is sometimes difficult to differentiate between amplitude and frequency effects as pulses become undetectable if the amplitude is highly suppressed. In the present study, there was an immediate and complete absence of LH pulses following icv infusion of the antagonist in 4 of the 6 animals, with the 2 remaining animals showing continuing LH pulses with a tendency for lower amplitude (though not significant) and with a gradual reduction in frequency. In addition, there was a rapid restoration of LH pulses with normal pulse amplitude but reduced frequency post-treatment. These findings suggested that kisspeptin affects the GnRH pulse generator. However, the most definitive evidence that the kisspeptin antagonist modulates frequency of the GnRH pulse generator was derived from our demonstration that intra-ARC administration reduced LH pulse frequency in a dose-dependent manner without affecting pulse amplitude. Interestingly, LH pulses were not affected by kisspeptin antagonist administration into the mPOA. This differential effect of antagonist administration into different sites of kisspeptin neuron localization suggests that the site of action of kisspeptin in controlling pulsatile LH secretion resides in the ARC nucleus and not the mPOA.

The present study has provided the first direct evidence that endogenous kisspeptin-GPR54 signalling in the ARC plays a critical role in regulating LH pulse frequency in the rat. It is well established that pulsatile LH secretion is governed by the GnRH pulse generator, the precise neural construct of which still remains to be elucidated. However, it has been proposed that kisspeptin neurons in the ARC nucleus may represent the substrate of the GnRH pulse generator [22], [23]. The GnRH pulse generator is thought to be located in the ARC nucleus of the mediobasal hypothalamus of the rat since isolation of this region, using the Halász deafferentation technique, permits continued follicular growth [24] and detection of LH pulses [25]. Further, the hypothalamic multiunit electrical activity (MUA) volleys invariably associated with LH pulses and which provide a robust electrophysiological correlate of GnRH pulse generator activity, are recorded from the ARC in the rat [20], [26] and goat [22]; species in which this structure contains abundant kisspeptin neurons but is devoid of GnRH neurons. It is worth noting that we recently reported that intravenous administration of kisspeptin-10 elicits a robust release of LH, and presumably GnRH, without affecting the MUA volleys in the rat [20]; data that are seemingly inconsistent with the notion that the MUA volleys reflect neuronal discharge recorded from GnRH neurons *per se*. Interestingly, Plant and Ramaswamy [23] have recently highlighted that the early ARC nucleus lesioning studies in the rhesus monkey that led Knobil to conclude that the GnRH pulse generator was located in this hypothalamic region [17], overlapped with the now known location of the kisspeptin perikarya [27]. The majority of the kisspeptin neurons in the ARC would therefore have been destroyed by the lesions, but the more laterally positioned GnRH neural network would have been spared [27]. Although the electrode assembly used for the MUA recordings in the monkey were targeted to area which kisspeptin neurons are located [27], [28], they would also have encompassed the GnRH neurons [29], so the neurochemical phenotype of the neurons that constitute these electrophysiological correlates in the monkey still remain an intriguing mystery. Nevertheless, Keen et al., [30] recently demonstrated a circhoral pattern of kisspeptin release showing 75% concordance with GnRH pulses simultaneously measured in the stalk-median eminence in the rhesus monkey, and that delivery of kisspeptin antagonist into the same region suppressed GnRH pulses measured in the stalk-median eminence dialysate [9]. Kisspeptin and its receptor are not only expressed in the ARC but their expression here is regulated by various physiological or pharmacological manipulations. For example, lactating rats not only show a profound suppression of pulsatile LH secretion, but also show a marked reduction in *Kiss1* mRNA expression [31]. Similarly, we have shown a down-regulation of *Kiss1* and GPR54 expression in the ARC in response to a variety of different stressors and corticotropin-releasing hormone administration, which are associated with suppression of LH pulses [32]. Collectively, these findings support a role for ARC kisspeptin-GPR54 signalling in the regulation of GnRH pulse generator activity.

Whether kisspeptin neurons in the ARC or indeed other loci are the key neural substrate of the GnRH pulse generator *per se* remains to be established. However, with clinical observations that patients with inactivating mutations of GPR54 exhibit low amplitude LH pulses with approximately normal frequency [2], [33], it remains equivocal whether kisspeptin neuron input is critical for the generation of pulsatile GnRH secretion or simply reflects modulation of GnRH pulse amplitude [9]. Furthermore, there is evidence that GnRH neurons themselves may be equipped with a pulse generating mechanism. Intrinsic periodic increases in intracellular calcium concentration synchronized with pulses of GnRH was detected in GnRH neurons from the olfactory placode of monkeys [34]. Episodes of spontaneous burst firing have also been detected in green fluorescent protein identified GnRH neurons [35].

The role of AVPV kisspeptin in GnRH surge generation is unequivocal. These kisspeptin neurons become transcriptionally activated at the time of the LH surge in the rat [36]. Local injection of kisspeptin into the preoptic area induced a surge-like increase in LH level in rats [10]. More importantly, infusion of a specific monoclonal antibody to kisspeptin into the preoptic area blocked the LH surge [10]. Whether the preoptic area kisspeptin-GPR54 signaling also contributes to GnRH pulse generation is unlikely given that in the present study intra-mPOA administration of the kisspeptin antagonist affected neither LH pulse amplitude nor frequency. It is of note that intra-mPOA administration of kisspeptin-10 evoked a robust release of LH, thus it can be presumed that the antagonist delivered into this region also has access to the kisspeptin receptor.

It is generally considered that kisspeptin directly stimulates GnRH neurons by binding to its cognate receptor on the perikarya. This was supported by earlier reports that both icv and peripheral administration of kisspeptin potently increased gonadotropic hormone secretion; an action accompanied by induction of c-FOS activation in GnRH neurons [13], [37]. More recently, electrophysiological studies using green fluorescent protein identified GnRH neurons in mice have shown exquisite sensitivity to kisspeptin with sustained action potential firing [38], [39]. Further, kisspeptin antagonist was found to block kisspeptin-10 stimulation of GnRH neuron firing [9]. However, in contrast to rodents there are few kisspeptin-GnRH cell body appositions in other species such as the rhesus monkey [27]. Nevertheless, there is increasing appreciation that kisspeptin may also act at the level of the GnRH nerve terminals, where there is close intermingling between kisspeptin and GnRH fibers at the level of the median eminence evident in many species including the goat [22], sheep [40] and rhesus monkey (27). Most recently, kisspeptin was shown to stimulate GnRH release, in a GPR54-dependent manner, from mouse mediobasal hypothalamus explants that contain GnRH nerve terminals but lack GnRH cell bodies [41]. Whether the marked stimulation of LH secretion following intra-ARC administration of kisspeptin-10, as previously shown for kisspeptin-54 [42], represents a possible local intranuclear effect involving an autoreceptor mediated positive feed-forward mechanism within the ARC kisspeptin neural network remains to be examined. Similarly, the dense plexus of kisspeptin cell bodies and fibers in the ARC [10] and evidence of close contacts between these fibers and cell bodies [43] may provide the anatomical substrate for locally administered kisspeptin antagonist to modulate LH pulse frequency. Although we cannot rule out the possibility that the intra-ARC administration of kisspeptin antagonist did not reach the median eminence to exert an effect at the level of the GnRH nerve terminals, there was no evidence of the dye injection, used to confirm cannula placement, reaching this structure. It is also difficult to provide an explanation for the apparent greater effect of the kisspeptin antagonist on suppression of LH pulse frequency and the tendency to lower LH pulse amplitude (in 2/6 animals) following icv administration compared with intra-ARC delivery. Whether this might reflect an action of the antagonist at the level of the median eminence following icv infusion [44] that may not occur after intra-ARC administration remains a possibility. However, other possible factors including continuous versus intermittent delivery of antagonist, or the difference in dosage of antagonist used for the icv versus intra-ARC may contribute to different efficacy.

It was recently reported that GnRH neuron specific expression of GPR54 in GPR54-null mice resulted in a complete rescue of fertility [45], which might suggest that kisspeptin-GPR54 signalling in the rodent ARC *per se* is non-essential for control of GnRH secretion. It is difficult to reconcile these data with the present findings of a modulatory influence of intra-ARC administration of the kisspeptin antagonist on GnRH pulse generator frequency, unless the antagonist was acting on GnRH nerve terminals in the median eminence and the latter region a locus for modulation of GnRH pulse frequency; a notion not inconsistent with the earlier findings of Rasmussen [46] of episodic GnRH release from the rat isolated median eminence. Further work is required to establish the precise site of action following intra-ARC administration of kisspeptin antagonist. Furthermore, there is always the possibility that the kisspeptin antagonist may be acting through mechanisms other than GPR54 receptor blockade, although there is considerable evidence against this notion [9].

In summary, the present study shows that intra-ARC, but not intra-mPOA administration of a selective kisspeptin antagonist reduced LH pulse frequency in a dose-dependent manner without affecting pulse amplitude in the rat. These data are the first to identify the arcuate nucleus as a key site for kisspeptin modulation of LH pulse frequency, supporting the notion that kisspeptin-GPR54 signalling in this region of the mediobasal hypothalamus is a critical neural component of the hypothalamic GnRH pulse generator.
